# Morphomolecular Characterization of Serum Nanovesicles From Microbiomes Differentiates Stable and Infarcted Atherosclerotic Patients

**DOI:** 10.3389/fcvm.2021.694851

**Published:** 2021-08-05

**Authors:** Camila Rodrigues Moreno, José Antonio Franchini Ramires, Paulo Andrade Lotufo, Alexandre Matos Soeiro, Luanda Mara da Silva Oliveira, Renata Nishiyama Ikegami, Joyce Tiyeko Kawakami, Jaqueline de Jesus Pereira, Marcia Martins Reis, Maria de Lourdes Higuchi

**Affiliations:** ^1^Laboratorio de Patologia Cardiaca, Instituto do Coracao, Hospital das Clinicas HCFMUSP, Faculdade de Medicina, Universidade de São Paulo, São Paulo, Brazil; ^2^Instituto do Coracao, Hospital das Clinicas HCFMUSP, Faculdade de Medicina, Universidade de São Paulo, São Paulo, Brazil; ^3^Hospital Universitario, Universidade de São Paulo, São Paulo, Brazil; ^4^Laboratório de Investigação em Dermatologia e Imunodeficiências - LIM56, Departamento de Dermatologia, Hospital das Clinicas HCFMUSP, Faculdade de Medicina, Universidade de São Paulo, São Paulo, Brazil

**Keywords:** myocardial infarction, extracellular vesicles, microbiome, archaea, *Mycoplasma pneumoniae*

## Abstract

Microbial communities are considered decisive for maintaining a healthy situation or for determining diseases. Acute myocardial infarction (AMI) is an important complication of atherosclerosis caused by the rupture of atheroma plaques containing proinflammatory cytokines, reactive oxygen species, oxidized low-density lipoproteins (oxLDL), damaged proteins, lipids, and DNA, a microenvironment compatible with a pathogenic microbial community. Previously, we found that archaeal DNA-positive infectious microvesicles (iMVs) were detected in vulnerable plaques and in the sera of Chagas disease patients with heart failure. Now, we characterize and quantify the levels of serum microbiome extracellular vesicles through their size and content using morphomolecular techniques to differentiate clinical outcomes in coronary artery disease (CAD). We detected increased numbers of large iMVs (0.8–1.34 nm) with highly negative surface charge that were positive for archaeal DNA, *Mycoplasma pneumoniae* antigens and MMP9 in the sera of severe AMI patients, strongly favoring our hypothesis that pathogenic archaea may play a role in the worst outcomes of atherosclerosis. The highest numbers of EVs <100 nm (exosomes) and MVs from 100 to 200 nm in the stable atherosclerotic and control healthy groups compared with the AMI groups were indicative that these EVs are protective, entrapping and degrading infectious antigens and active MMP9 and protect against the development of plaque rupture.

**Conclusion:** A microbiome with pathogenic archaea is associated with high numbers of serum iMVs in AMI with the worst prognosis. This pioneering work demonstrates that the morphomolecular characterization and quantification of iEVs in serum may constitute a promising serum prognostic biomarker in CAD.

## Introduction

Microbial communities have been described in humans and are considered decisive for maintaining a healthy situation or determining diseases (1–3). The composition of these microbial communities is affected by external stimuli from both the host and other symbiotic pathogens. Changes in the composition of the microbial community are associated with the worsening of several diseases, such as atherosclerosis, diabetes mellitus, hypertension, obesity, and heart failure (4, 5).

Acute myocardial infarction is an important complication of atherosclerosis that is usually caused by rupture of atheroma plaques (6, 7). An atherosclerotic plaque containing proinflammatory cytokines, reactive oxygen species, high levels of oxidized low-density lipoproteins (oxLDL), cholesterol crystals and damage-associated molecular patterns (8), due to damaged proteins, lipids and DNA (9), is a microenvironment at risk for plaque rupture and thrombosis (10), and might be explained by the presence of a pathogenic microbial community (11). In previous work by our group, the presence of archaea was related to the aggravation of atherosclerosis (12, 13) and with heart failure in Chagas disease, where archaeal DNA was detected in nanoparticles in the serum (14).

Archaea are the oldest microorganisms existing in nature and are resistant to bacterial antibiotics, featuring a negatively charged membrane, oxidation capacity, production of metallopeptidases (15), and induction of inflammation through the activation of TCD8+ lymphocytes (16), a characteristic also present in chagasic myocarditis (17).

Another factor isolated from atherosclerotic plaques that contributes to AMI is the presence of highly thrombogenic extracellular vesicles (EVs) (18–20). EVs have emerged as a marker in several diseases, such as cancers and cardiovascular diseases, through their quantification and characterization (21–23). Their biological properties provide important information since they can participate in intercellular communication. According to size, EVs are classified into microvesicles (MVs, 0.1–1 μm) and exosomes (Exos, <0.1 μm) and are present in most body fluids. They can be protective, promoting the removal of harmful cellular compounds leading to cell survival, or deleterious, favoring signaling mechanisms that are harmful to the heart, which can lead to the development of heart failure (24–26). In addition, the literature has described those infectious agents also release extracellular vesicles (iEVs), called infectious exosomes (iExos), or infectious microvesicles (iMVs) (27, 28). Here, we characterize and quantify the levels of serum microbiome extracellular vesicles through their size and content to evaluate differences in the clinical evolution of coronary artery disease patients.

## Materials and Methods

### Study Design and Patients

This study was carried out at the Heart Institute (InCor) of the University of São Paulo in São Paulo, Brazil, from September 2018 to March 2020. The study protocol and informed consent form (ICF) were submitted to the Scientific Committee of InCor and were approved by the Ethics Committee for Research Project Analysis (CAPPesq) of Hospital das Clínicas, Faculty of Medicine, University of São Paulo (HCFMUSP), under protocol numbers 029/04 and SDC 2356/03/150. We prospectively studied 168 patients with atherosclerotic disease and ST segment infarction (STEMI) and healthy individuals. STEMI patients were divided into groups according to their disease severity following the Killip classification (29), which has already been consolidated in the clinic. The classification was performed at the time of admission to the emergency room and was determined for each patient by the doctor who admitted it. The eligibility criteria and the sample composition of the different study groups are described below:

- AMI (*n* = 40): (AMI grade Killip I or II, with ST elevation by ECG).
- Severe AMI (*n* = 34): (AMI grade Killip III or IV, with ST elevation by ECG).
- CTL (*n* = 34): (Control, healthy individuals (LDL <130, SAH <130/80 and blood glucose <99)
- ATR (*n* = 40): (Atherosclerotic, with obstruction ≥70% in at least two main arteries observed by coronary angiography).

To complete the cases of the severe AMI and CTL groups, we used sera from 10 patients from the longitudinal clinical studies ELSA (Brazilian Longitudinal Study of Adult Health) and ERICO (Strategy of Registry of Acute Coronary Syndrome), with approval by CaPPesq from the University Hospital of the University of São Paulo (protocol: 866/08F and SDC: 2356/03/150).

### Separation and Isolation of Microvesicles and Exosomes

In this study we used a new method for isolating extracellular vesicles. Approximately 10 ml of whole blood was collected in a dry tube from patients in the different groups at the time of admission. Subsequently, the tubes were left for 1 h at 37°C to promote clot retraction. Then, they were centrifuged at 2,000 rpm for 10 min, and sera were aliquoted in microtubes and stored in a freezer at −80°C. The following isolation method was performed according to a mitochondria separation procedure described by Bustamente et al. (30) and modified by us, which enabled a high recovery of microvesicles and serum exosomes. The sera were diluted (1:5) in H medium containing 200 mM D-mannitol (Fresenius Kabi Brasil, Aquiraz, CE, BR), 70 mM sucrose (EMS Electron Microscopy Sciences—Hatfield, PA, USA), 2 mM HEPES (Fair Lawn, NJ, USA), and 0.5 g/L BSA pH 7.2 (Sigma, St. Louis, MO, USA) and incubated for 1 h at room temperature (RT). The samples were then centrifuged for 12 min at 9,500 × g, and the supernatants were collected for processing by transmission electron microscopy (TEM).

### Validation and Comparison of Isolation Methods Using Sucrose-Rich Medium

ISEV (International Society of Extracellular Vesicles) claims that so far there is no consensus on a “gold standard” method for isolating and/or purifying EVs, they recommends that the researcher chooses the most efficient method to answer their scientific question. Therefore, we chose this method, which has a medium rich in sucrose and manitol similar with the density and osmolarity of media and used in studies with isolation by differential ultracentrifugation (dUC), able to maintaining EVs with similar density to this method already validated by the ISEV (31). The dUC procedure begins with a series of spin cleanup steps designed to remove cells, cell debris, apoptotic bodies, and microvesicles. This is done by gradually separating the pellet and supernatant at increasing speeds: 300–400 × g for 10 min, then 2,000 × g and finally 10,000 × g, equivalent to the rotation used by us to isolate a supernatant containing a high concentration of exosomes, although still “contaminated” with microvesicles. After this step, the final sedimentation to obtain the purified exosome population occurs by rotating the samples at 100,000–200,000 × g for 2 h or at least 70 min (32). However, our study aims not only to characterize but to quantify distinct populations of particles (exosomes, small and large microvesicles, and infectious EVs) in different groups for stratification of atherosclerotic CAD. The purification to obtain a specific population would lead to loss of a sample with heterogeneous populations, which may lose important information about the difference both in the composition and in the concentration of the different subtypes of EVs according to the severity of the disease. Furthermore, it is known that the application of ultracentrifuge force can change the morphology of EVs (33).

Because of the problems and reasons presented, we used a medium that is equivalent in density to the widely used sucrose-rich medium, and applied the required force (~10,000 g) already validated to obtain a sample with a high concentration in exosomes but still have other microvesicles present. Despite being a comparable method, it has not yet been validated by ISEV, so we will demonstrate that this isolation method is effective through immunostaining for a specific exosome marker (CD63) by the Western Blotting technique.

### Morphological Characterization of EVs by TEM

#### Fast Inclusion Method

Inclusion was performed following a quick procedure described by Duarte et al. (34), modified by us as described below: After the separation phase, to detect exosomes present in the supernatant, 400 μl of the supernatant fraction was fixed by the addition of 1 ml of 3% glutaraldehyde at 4°C for 3 h, post-fixed by the addition of reduced osmium solution (700 μl of 1% osmium tetroxide solution + 700 μl of 3% potassium ferrocyanide) at 4°C for 30 min and centrifuged at 13,000 × g for 5 min at 12°C. This procedure allowed the formation of the pellet because the exosomes and lipidic microvesicles present in the supernatant became heavier after fixation. Then, the supernatant pellets were washed in a solution containing 0.9% NaCl + 360 mOs of sucrose and incubated in 0.5% uranyl acetate for 3 h at 4°C. The pellets were dehydrated for 10 min in 70% ethanol, 5 min in 0.1 N acidified 2,2-dimethoxypropane with HCl, and incubated for 2 min in acetone with 4% copper sulfate. Infiltration was performed with a mixture containing epon EMbed 812 resin with araldite 502 resin (1:1) and polymerization in a 100°C oven for 1 h. The blocks were cut in an ultramicrotome with a thickness between 60 and 70 nm and were placed in copper and nickel grids of Parlodium 200 mesh (all reagents and grids were from Electron Microscopy Sciences, Hatfield, PA, USA).

#### iEVs Characterization by Electron Microscopy Immunolabeling

To remove the resin and expose the masked antigenic sites, grids were incubated in 0.5 M sodium metaperiodate solution (Sigma, St. Louis, MO, USA) for 1 min at RT, washed 4 times in distilled water and incubated in CAS-block (Life Technologies, Frederick, MD, USA) for 30 min at RT. They were then incubated with anti-MMP9 (rabbit polyclonal antibody, #RB1539P0, Thermo Scientific, UK) and *M. pneumoniae* (rabbit polyclonal antibody, #20MR54, Fitzgerald, USA) for 16 h at 4°C, washed in phosphate-buffered saline (PBS), and incubated for 1 h at RT with diluted secondary antibody labeled with 10 nm gold particles (EMS—Electron Microscopy Sciences, Hatfield, PA, USA). The grids were contrasted with 0.08 M lead citrate solution for 5 min according to the technique described by Reynolds (35) for later observation by TEM. Immunolabeling analysis by electron microscopy was performed in 25% of the total sample size.

#### Analysis and Distribution of the Size and Concentration of Serum EVs by Nanoparticle Tracking—NTA (NanoSight)

Nanoparticle tracking analysis (NTA) was performed using a NanoSight LM10 instrument coupled to a highly sensitive sCMOS camera (Malvern Instruments, Ltd., Malvern, UK), which captures a video file of the serum EVs moving under Brownian movement. EVs isolated from patient serum were diluted (1:50) in particulate-free H medium (filtered at 0.02 μm) to obtain a concentration within the recommended medication range (1–10 × 10^8^ particles/mL). The following settings were used: camera level set to automatic, threshold and focus set manually to optimize readings according to the manufacturer's instructions, a measurement temperature of 25°C, 5 runs of 30 frames per second and a time measurement time of 60 s. The size distribution and concentration of EVs were analyzed using NTA v.3.4 software (Malvern Panalytical, Ltd., Malvern, United Kingdom), applying the Strokes-Einstein equation. Absolute quantification of EVs by NTA method was performed in 50% of the total sample size.

#### Western Blotting

Total protein from supernatant enriched with extracellular vesicles was extracted by using an extraction RIPA buffer (25 mM Tris-HCl pH 7.6, 150 mM NaCl, 1% NP-40, 1% sodium deoxycholate, 0.1% SDS) containing protease and phosphatase inhibitors. After centrifugation (21,000 g, 15 min, 4°C), the supernatant was stored at −80°C. Protein concentration was determined by Bradford's method (36) and was calculated according to the line equation obtained from a concentration curve. Thirty microgram of total protein was incubated with Laemmli buffer and boiled for 5 min. Samples were electrophoresed (140 V for 1 h), transferred onto a nitrocellulose membrane using a Mini Trans-Blot Cell (100 V for 1 h; Bio-Rad) and incubated with primary antibody (RT for 4 h). The following antibodies were used: CD63 (#ab134045; Abcam); β-actin (#sc47778; Santa Cruz). Membranes were incubated with peroxidase-conjugated secondary antibody (RT for 1 h) and submitted to a chemiluminescent reaction (Luminata Forte Western HRP Substrate, Millipore). The protein band was analyzed by a transilluminator (UVItec Limited, Cambridge).

#### Analysis of the Enzymatic Activity of MMP9 by Gelatin Zymography

This procedure was used to determine the levels of active MMP9 in samples of supernatant serum from infarcted, atherosclerotic patients and the control group. Approximately 100 μg of protein from each sample previously extracted and dosed was submitted to polyacrylamide gel electrophoresis. The samples were incubated with Tris-glycine SDS Novex™ 2x Buffer (Thermo Fisher) without the presence of a reducing agent or heating to maintain the function of the enzymes present. All samples were loaded on a 10% polyacrylamide gel together with 4 mg/ml gelatin (G9391 Sigma) since it is a protease substrate specific to gelatinase-class enzymes. After electrophoresis, the gel was washed with 1X renaturation buffer containing non-ionic detergent and 2.5% Triton X-100 (Novex Zymogram Renaturation Buffer—Invitrogen) for 30 min at RT under gentle agitation. Subsequently, the gel was incubated in Tris buffer for 30 min at RT. This incubation was repeated for 48 h at 37°C, allowing the digestion of the substrate by the protease. The gel was washed with deionized water, stained for 1 h with Coomassie Blue R-350, and placed in a plastic support of 300 dpi or higher. The intensities of the bands were determined by densitometry using the UVITEC Alliance Q4-365 Advanced Imaging System software. The digested clear bands were normalized to β-actin protein values, and each gel contained samples of AMI, severe AMI, ATR, and CTL samples whose values were considered 100% and served as a basis for quantifying the other two groups.

#### Determination of the EVs Electrical Charge by the Zeta Potential

In an attempt to understand whether archaea linked to the AMI group had different characteristics from the other EVs, the electrical charge was determined by measuring the zeta potential (electrical potential) of each sample using the electrophoretic light scattering technique on a ZetaPals device (Brookhaven Inst. Corp.). All samples were analyzed using a helium-neon laser (29 mW, l = 658 nm) to excite the samples under an angle of detection of the scattered light fixed at 90°C at a temperature of 25°C in acrylic cuvettes. The supernatants were diluted 100 times in H medium (200 mM D-mannitol, 70 mM sucrose, 2 mM HEPES, and 0.5 g/L BSA, pH 7.2) and filtered through a 0.22-μm nitrocellulose membrane to remove “contaminants” and remnants of salts that could interfere with the determination of the electrical charge of the EVs.

#### Analysis of iMVs by Flow Cytometry

Microvesicles in the serum were also quantified in a flow cytometer using a procedure adapted from previously described protocols (37, 38) by calibration with polystyrene microspheres of defined size (0.8–1.34 μm Spherotech Inc., Libertyville, IL, USA) which has properties comparable to EVs were used to define the limits of the detection area.

Hybridization was performed for archaeal DNA using a generic biotinylated probe, ARCH 915 (GTGCTCCCCCGCCAATTCCT). Each 20-μl sample of serum was incubated with the probe (40 ng/μl) in an oven at 45°C for 20 h and then incubated with PE Texas Red Streptavidin (BD Pharmingen^TM^, USA) for 30 min at RT, protected from light. The archeal DNA probe was previously diluted to 400 ng/ml in hybridization buffer (50% formamide, 10X saline-sodium citrate buffer [SSC: 1.5 M NaCl, 0.15 M sodium citrate, pH 7], 2X Denhardt's solution, 200 μg/mL salmon sperm DNA and 10% dextran sulfate). This buffer allows hybridization to occur with a high amplification, forcing the probe and target into close proximity and blocking binding to non-specific targets.

The identification of antigens from MVs was performed by incubating each serum with specific antibodies for *Mycoplasma pneumoniae* and MMP-9 collagenase using dilutions of 1:400 and 1:1,000, respectively. The samples were incubated with the primary antibodies for 1 h at RT and were then incubated with secondary antibodies (AlexaFluor 488 and 555 Invitrogen, CA) for 30 min at RT in the dark. Additionally, mouse IgG antibodies labeled with FITC and PE (isotype control) were used. The immunolabeled microvesicles were quantified after the acquisition of 10,000 events using a flow cytometer (BD LSRFortessa™, Becton Dickinson, USA) and analyzed with FlowJo software (Tree Star).

#### Analysis of the Inflammatory Profile by Cytometric Bead Array

Measurements of cytokines in the total sera of patients in the different groups were performed using the cytometric bead array (CBA) method, using the Human Inflammation Enhanced Sensitivity Flex Set kit (IL-2, IL-10, IL-6, IL-1β, IFN-γ, and TNF) (BD Pharmingen, CA, USA). The sera and the cytokine standards from the kit were incubated with capture microspheres covered with specific antibodies for the respective cytokines and with the detection antibody labeled with phycoerythrin (PE). After the incubations, 1 mL of the washing solution was added and centrifuged for 10 min at 1,100 rpm. The supernatant was discarded, and 300 μL of the washing solution was added. The samples were resuspended for acquisition in a BD LSRFortessa™ (Becton Dickinson, USA). The results were generated in graphic format and tabulated using BD CBA Analysis Software.

#### Statistical Analysis

The software used in the statistical calculations was GraphPad Prism 6. The data for each variable were initially compared with the normal curve by the Kolmogorov-Smirnov distance test and were classified as parametric or non-parametric. Parametric data are described as the mean ± standard deviation, and the groups were compared by analysis of variance (ANOVA), with subsequent application of the Tukey post-test. Non-parametric data are described as medians and interquartile ranges, and the groups were compared using the Kruskal-Wallis test with Dunn's *post-hoc* test. Values of *p* < 0.05 were considered statistically significant.

## Results

### Studied Populations—Baseline Characteristics

We observed that the patients in the ATR group were older (mean 70.23 years), and 72% were male. Conversely, the patients in the AMI group had a lower mean age (55.7 years), and a higher percentage were male (75%). The mean age of patients with severe AMI was 68.50 years, with 50% being male and the other half being female. The CTL group was composed of individuals with an average age of 51.50 years old, with 67.64% being male. With regard to risk factors, patients in the ATR group were those who had the highest percentages of diabetes, hypercholesterolemia, hypertriglyceridemia, and hypertension and had higher BMIs. An interesting fact is that patients with severe AMI had a mortality rate of 47.05%, and the evolution of death occurred before 30 days after the event. In addition, the group with severe AMI had the highest percentages of diabetic and hypertensive patients.

The characteristics of the baseline population are found in the Supplementary Table 1.

### Infarcted Patients Have Fewer Total Circulating EVs in Serum Than Non-infarcted Individuals—Absolute Quantification by NTA

**Figure 1:**
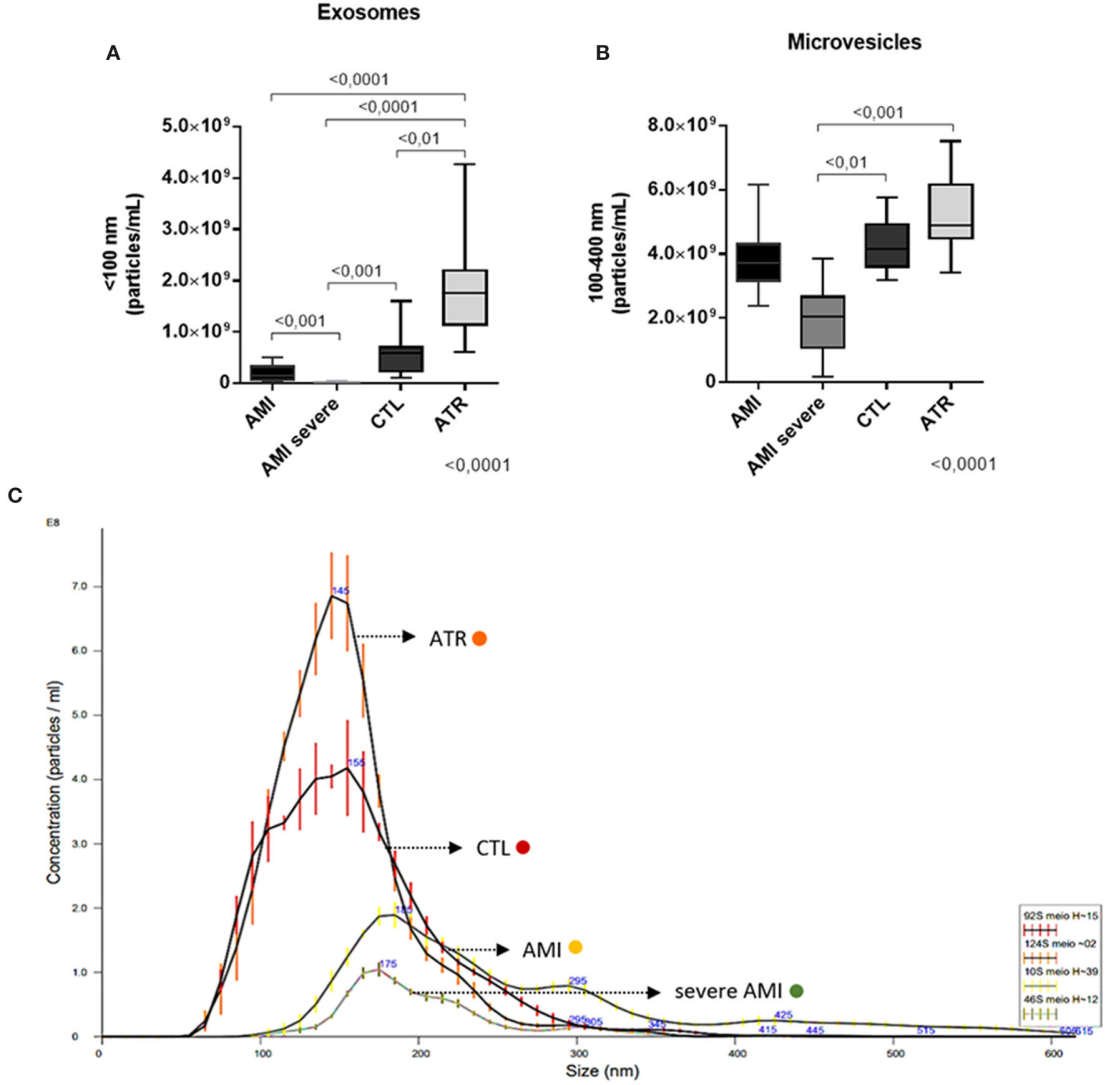
**(A,B)** Absolute quantification (particles/mL) of serum exosomes (<100) and microvesicles (100–400 nm). The decrease in circulating exosomes is accompanied by the progression of worsening evolution. In exosomes, the decrease occurs more significantly (*n* = 20. per group). **(C)** Representative curve of the concentration and size dispersion of the total serum EVs in the different study groups (orange, ATR; red, CTL; yellow, AMI; green, severe AMI). (Data are expressed as the mean ± standard deviation). Comparison by Kruskal-Wallis with Dunn applied as a post-test.

The absolute quantification of EVs showed higher numbers of Exos in the ATR [1.76^9^ (1.l3^9^−2.19^9^)] than in the AMI [1.40^8^ (5.70^7^−3.25^8^)], severe AMI [8.50^6^ (7.50^5^−1.06^7^)], and CTL [5.79^8^ (2.22^8^−7.02^8^)] groups (Kruskal-Wallis, *p* = 0.0001). The severe AMI group presented the lowest number of Exos (Figure 1A). The numbers of MVs in the severe AMI group [2.12^9^ (1.51^9^−2.83^9^)] were lower than those in the CTL [3.9^9^ (3.65^9^−4.62^9^)] and ATR [5.85^9^ (4.83^9^−6.90^9^)] groups. In addition, the AMI group [3.58^9^ (3.16^9^−4.20^9^)] also exhibited fewer microvesicles than the ATR group (Figure 1B). Through the concentration and size dispersion curve of the total serum EVs in the different study groups, it is possible to see that ATR and CTL groups have the largest amount of both particles as a whole and the small ones (<100 nm) referring to exosomes, in relation to the AMI and severe AMI groups (Figure 1C).

### Severely Infarcted Patients Have High Amounts of Circulating MMP9 and *M. pneumoniae* Antigens and Exosome Deficiency—Semi Quantitative Analysis by TEM

Quantification of Exos (<100 nm)/photo by TEM showed a lower number in the severe AMI group [15.5 (4–112.8)] than in the ATR [139.5 (28–327)] and AMI [172.5 (72–400)] groups (Kruskal-Wallis, *p* < 0.001). The AMI group also had a higher number than the CTL group [34.5 (8.25–219)].

**Figure 2:**
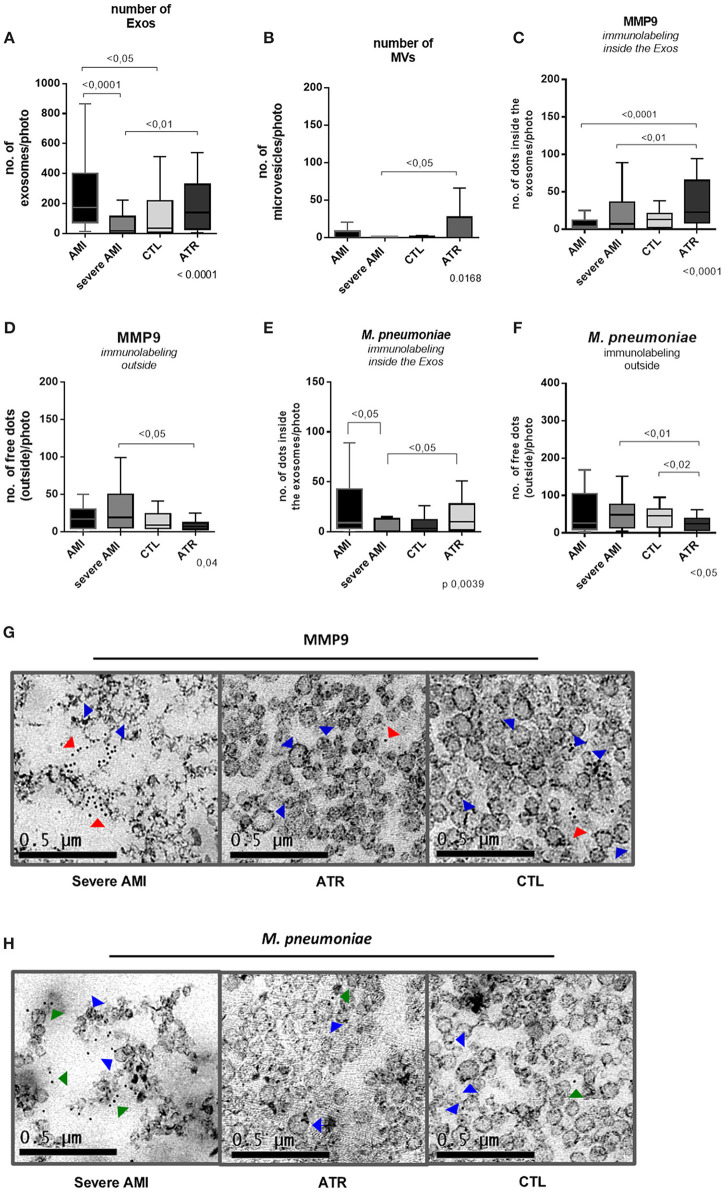
Immunolabeling for MMP9 and *M. pneumoniae* by transmission electron microscopy. The decrease in exosomes leads to poor removal of MMP9 and *M. pneumoniae* antigens in severe infarction. **(A)** Number of exosomes/photos. **(B)** Number of microvesicles/photo (*n* = 10. per group). **(C)** Number of MMP9 antigens (dots) inside exosomes/photo (*n* = 10. per group). **(D)** Number of MMP9 antigens (dots) outside exosomes/photo (*n* = 10. per group). **(E)** Number of *M. pneumoniae* antigens (dots) inside exosomes/photo (*n* = 10. per group). **(F)** Number of *M. pneumoniae* antigens (dots) outside exosomes/photo (*n* = 10. per group). **(G,H)** Severe AMI has a greater number of free antigens (red arrow, MMP9; green arrow, *M. pneumoniae*), and ATR has greater numbers of MMP9 and *M. pneumoniae* antigens within the EVs (blue arrow). Immunogold 10 nm −50,000X.

The quantification of MVs (>100 nm/photo) revealed a lower number in the severe AMI group [0.0 (0–0.75)] than in the ATR group [0.0 (0–27)] (Kruskal-Wallis, *p* < 0.01; Figures 2A,B).

Through immunolabeling by electron microscopy, there were fewer positive dots of MMP9 inside Exos/photo in the severe AMI [7.0 (0–36)] and AMI groups than in the ATR group [23 (8.5–65)] (Kruskal-Wallis, *p* < 0.0001). In addition, the severe AMI group [19 (5–15)] had a higher number of free antigens than the ATR group [7 (3–12)] (Kruskal-Wallis, *p* = 0.04; Figures 2C,D). Immunolabeling of *M. pneumoniae* revealed fewer positive dots inside Exos/photo in the severe AMI group [0.5 (0–13)] than in the ATR group [10 (1.5–27.5)] (Kruskal-Wallis, *p* = 0.0039). The group with severe AMI [48.5 (13.2–76.5)] also exhibited a greater number of *M. pneumoniae*-free antigens than the ATR group [24 (7.25–38.75)] (Kruskal-Wallis, *p* < 0.05; Figures 2E,F).

In representative images of the immunolabeling, it is possible to verify that the severe AMI group had a greater number of MMP9 and free *M. pneumoniae* antigens compared to the ATR group, which presented a high number of antigens within the EVs (Figures 2G,H).

### Validation of Extracellular Vesicle Separation Method—CD63 Exosome Specific Marker

**Figure 3:**
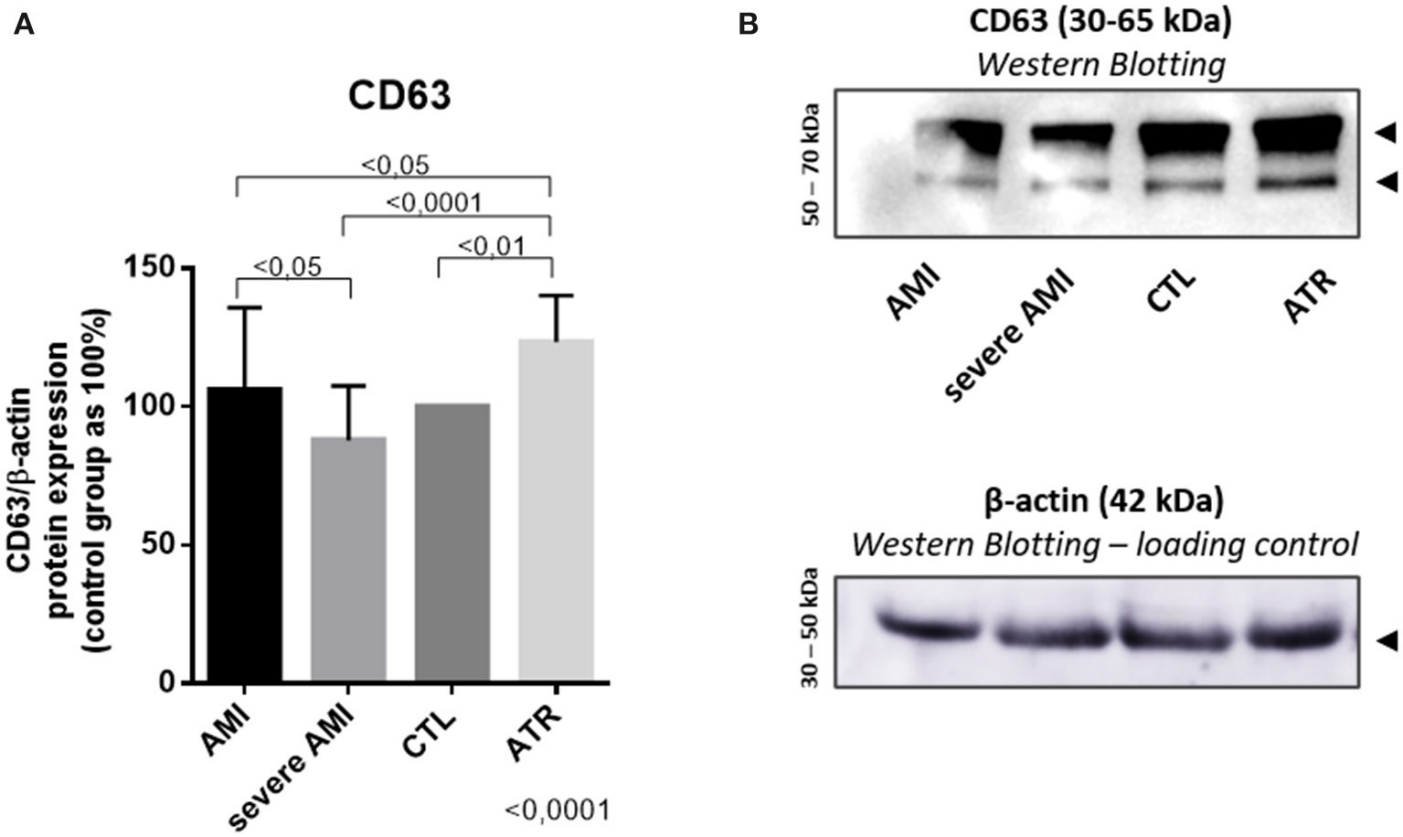
Separation method validation—CD63 exosome specific marker. CD63 protein expression are positively modulated in patients with better prognosis **(A)** Quantification of CD63 levels (%), with the control group set to 100% (*n* = 34. per group). **(B)** Representative gel of CD63 in the study groups. β-actin is used as a loading control. (Data are expressed as the mean ± standard deviation). Comparison by ANOVA with Tukey applied as a post-test.

The expression levels of exosome marker (CD63) were validated using WB analysis. The results indicated that this exosome biomarker showed higher in the ATR group (123.4 ± 16.79) than in the severe AMI (87.90 ± 16.65), AMI (106.1 ± 29.71), and CTL (100 ± 0.0) groups (ANOVA, *p* < 0.0001). The AMI group also exhibits increased expression compared to the severe AMI group (Figures 3A,B).

### Enzyme Activity of MMP9 Is Elevated in Infarcted Patients and Increases With the Severity of the Disease

**Figure 4:**
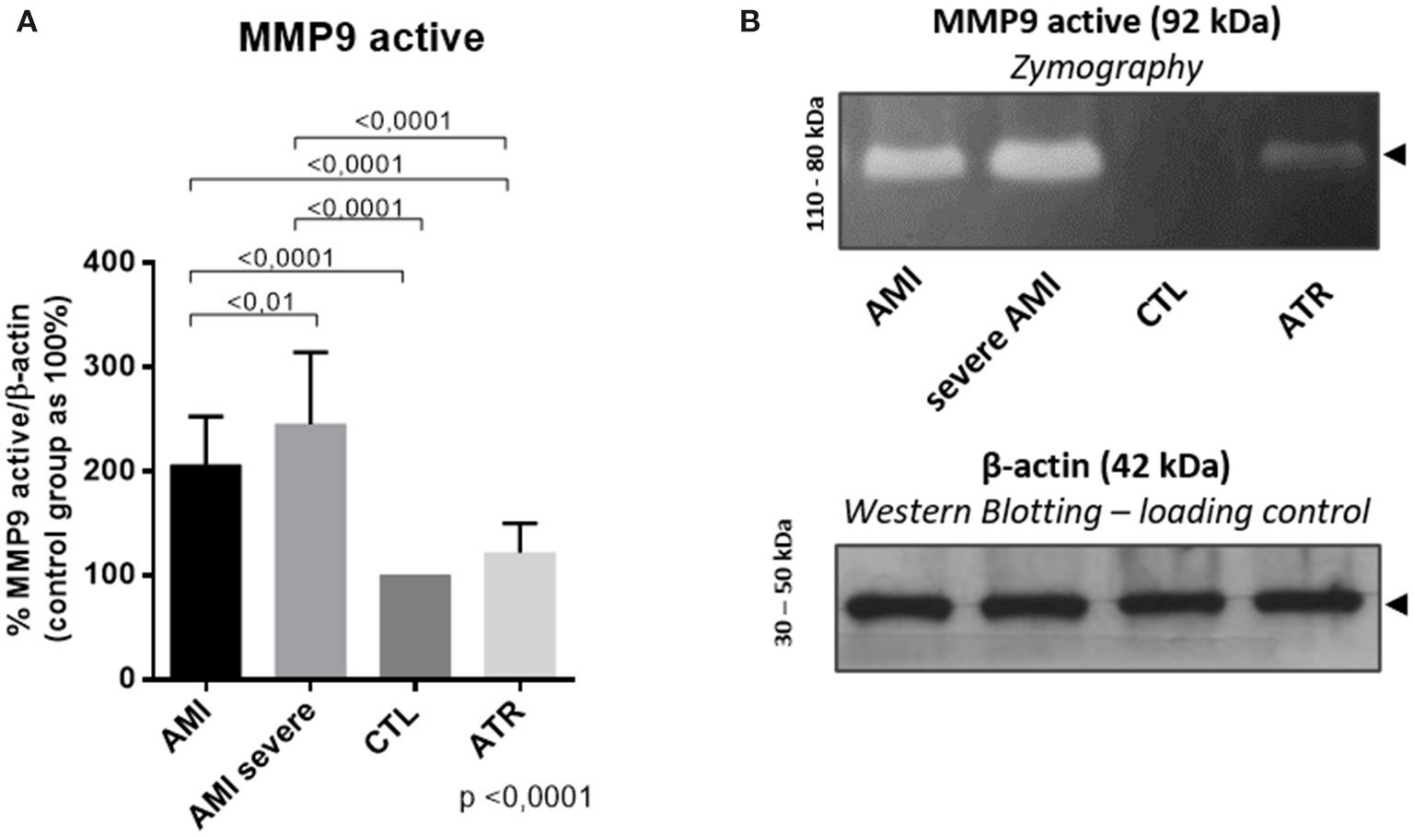
Analysis of the enzymatic activity of MMP9 through gelatin Zymography. There is positive regulation of the catalytic activity of MMP9 in infarcted patients, and the severity accentuates the activation. **(A)** Quantification of active MMP9 (%), with the control group set to 100% (*n* = 34. per group). **(B)** Representative gel of MMP9 activity in the study groups. β-actin is used as a loading control. (Data are expressed as the mean ± standard deviation). Comparison by ANOVA with Tukey applied as a post-test.

The zymography digestion band corresponding to the pro-enzymatic forms of MMP9 (Figure 4A) showed higher MMP9 activity in the AMI [203.4 (176.0–254.4)] and severe AMI [237.4 (207.2–301.9)] groups than in the CTL [100 (100–100)] and ATR groups [122.5 (108.9–142.4)] (ANOVA, *p* < 0.0001); the severe AMI group exhibited significantly higher MMP9 activity compared to the AMI group (Figures 4A,B).

### Higher Serum Concentration of Infectious Large Microvesicles (0.8–1.34 μm) in Patients With Severe Infarction

**Figure 5:**
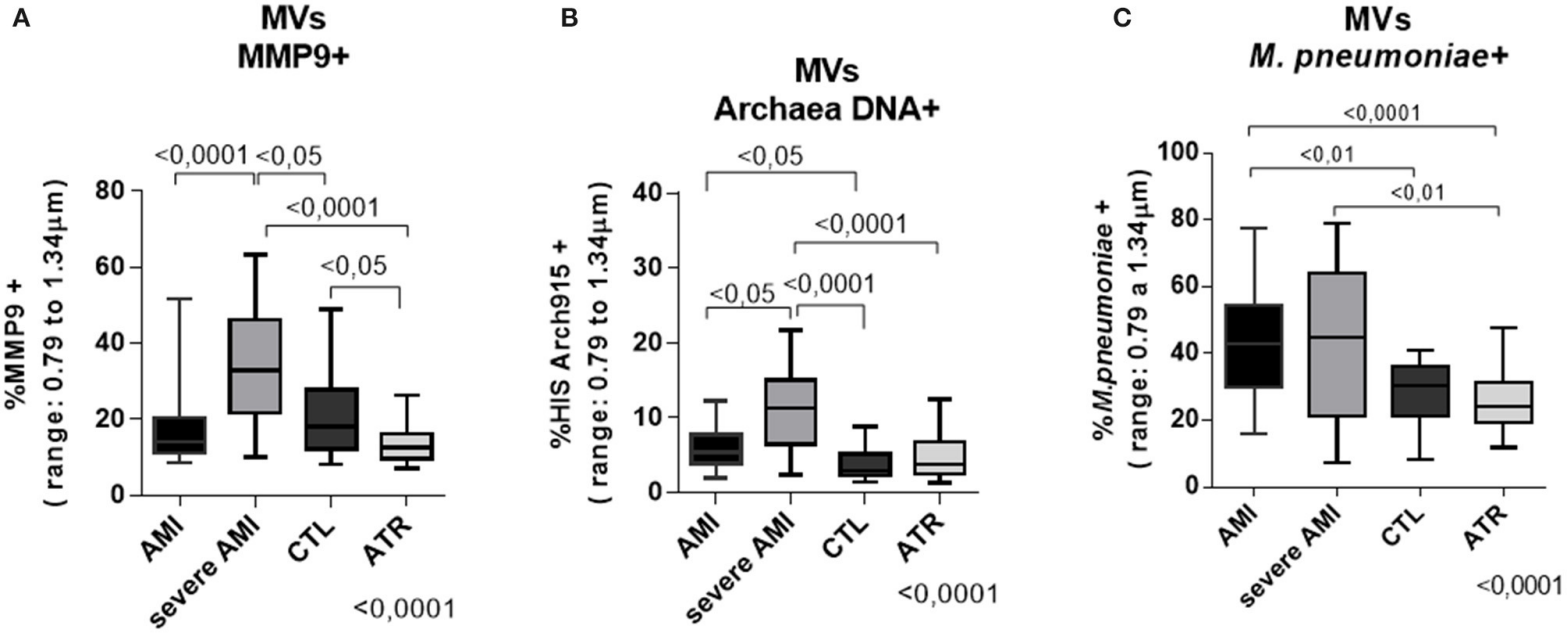
Quantification by flow cytometry. The amount of MVs of infectious agents increases progressively according to the severity of atherosclerotic disease, and the symbiosis observed between archaea and *M. pneumoniae* observed in infarcted patients increases the number of positive MMP9 MVs, especially in severe cases. **(A)** % MMP9-positive MVs **(B)** % MVs with archaeal DNA (Arch915 probe) **(C)** % *M. pneumoniae*-positive MVs (size: 0.8–1.34 μm—acquisition of 10,000 events) (AMI *n* = 40, severe AMI *n* = 34, CTL *n* = 34, ATR *n* = 40) (data are expressed as median and interquartile range 25–75%). Comparison by Kruskal-Wallis with Dunn applied as a post-test.

The numbers of MVs positive for MMP9 antigens and for archaeal DNA were higher in the severe AMI group [32.90 (11.20–20.28)] and [11.30 (6.46–15.05)] (median and interval interquartile 25–75%), respectively, compared to the AMI [14.05 (11.20–20.28)] and [5.41 (3.88–7.77)], ATR [12.55 (9.54–16.20)] and [3.77 (2.52–6.81)], and control [18 (12.03–27.7)] and [2.90 (2.31–5.13)] groups, respectively (Kruskal-Wallis, *p* < 0.0001; Figures 5A,B).

The percentage of MVs positive for *M. pneumoniae* antigens was also significantly higher in the AMI [42.84 (30.13–54.30)] and severe AMI [45 (21.15–64.05)] groups than in the ATR [24.15 (19.55–31.48)] and CTL [30.2 (21.25–35.98)] groups (Kruskal-Wallis, *p* < 0.0001). The severe AMI group also exhibited a greater number of positive MVs than the CTL group (Figure 5C).

### Zeta Potential Measurement: Presence of Archaea Decreases Electrical Charge of EVs

**Figure 6:**
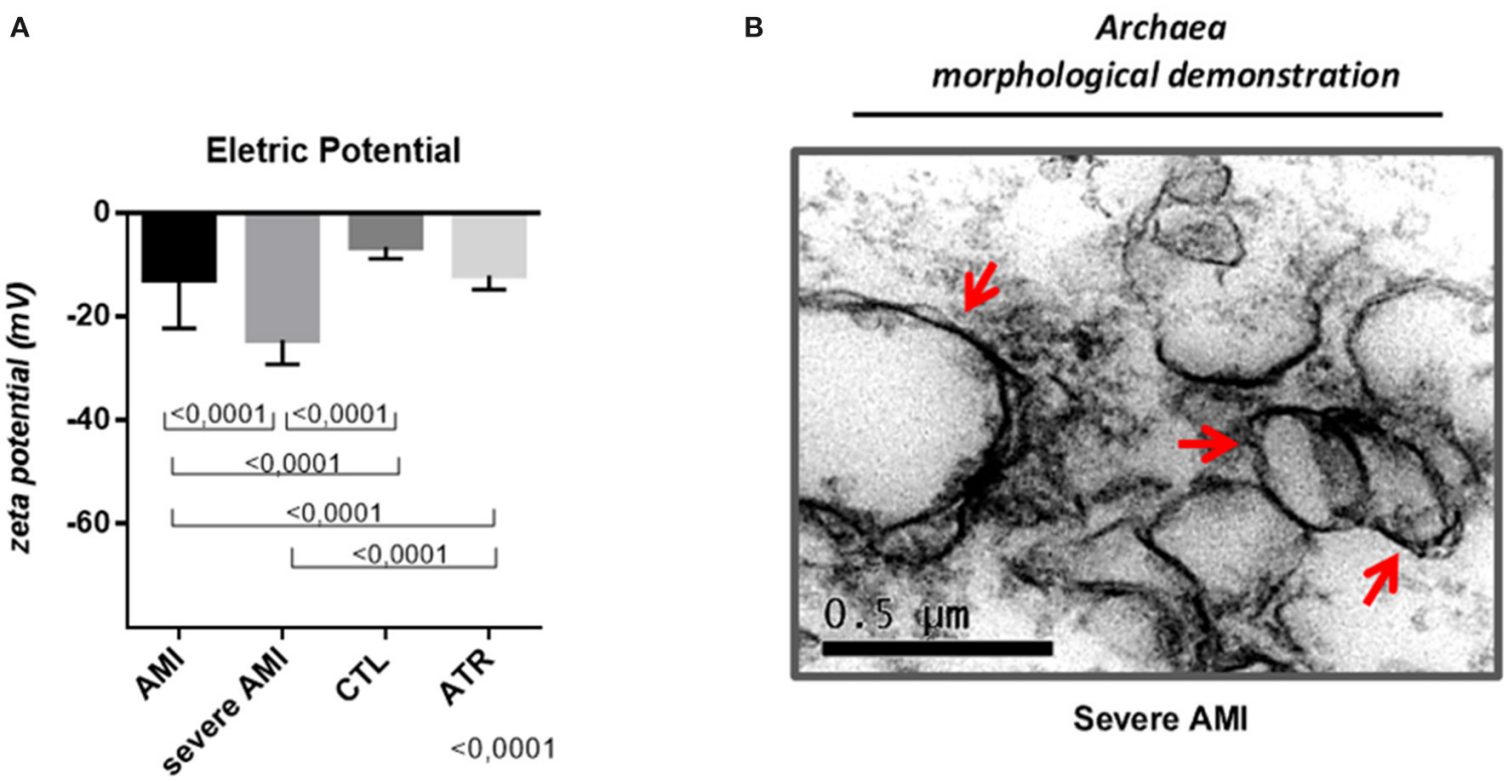
Quantification of the electrical charge of EVs—Zeta Potential. Decreased EVs electrical charge is accompanied by worsening of the evolution of atherosclerotic disease. **(A)** Infarcted patients, especially those who have a greater amount of pathogenic archaea, exhibit EVs with a highly negative electrical charge. EVs (Exos and MVs) with negative electric charge (mV) (AMI *n* = 40, severe AMI *n* = 34, CTL *n* = 34, ATR *n* = 40). **(B)** Demonstration of the morphological characteristic of archaea present in severe AMI. (Data are expressed as the mean and standard deviation). Comparison by ANOVA with Tukey applied as a post-test. Red arrows: Morphological Demonstration of Archaeas by Transmission Electron Microscopy.

The zeta electrical potential showed a greater number of EVs with a highly negative charge in the severe AMI group (−24.46 ± 4.73), which was significantly higher than those in the other groups [AMI (−12.79 ± 9.46), CTL (−6.46 ± 2.27), and ATR (−11.96 ± 2.71)] (ANOVA, *p* < 0.0001). The AMI group also had an increased negative electrical charge compared to the CTL and ATR groups (Figure 6A). Demonstration of MVs (severe AMI group) with double electro-negative membrane and electron lucent content, morphologically characterized as archaeas (Figure 6B).

### Inflammatory Cytokine Panel (CBA): High Circulating Concentration IL-6 and IL-10 in Infarcted Patients With Worse Prognosis

**Figure 7:**
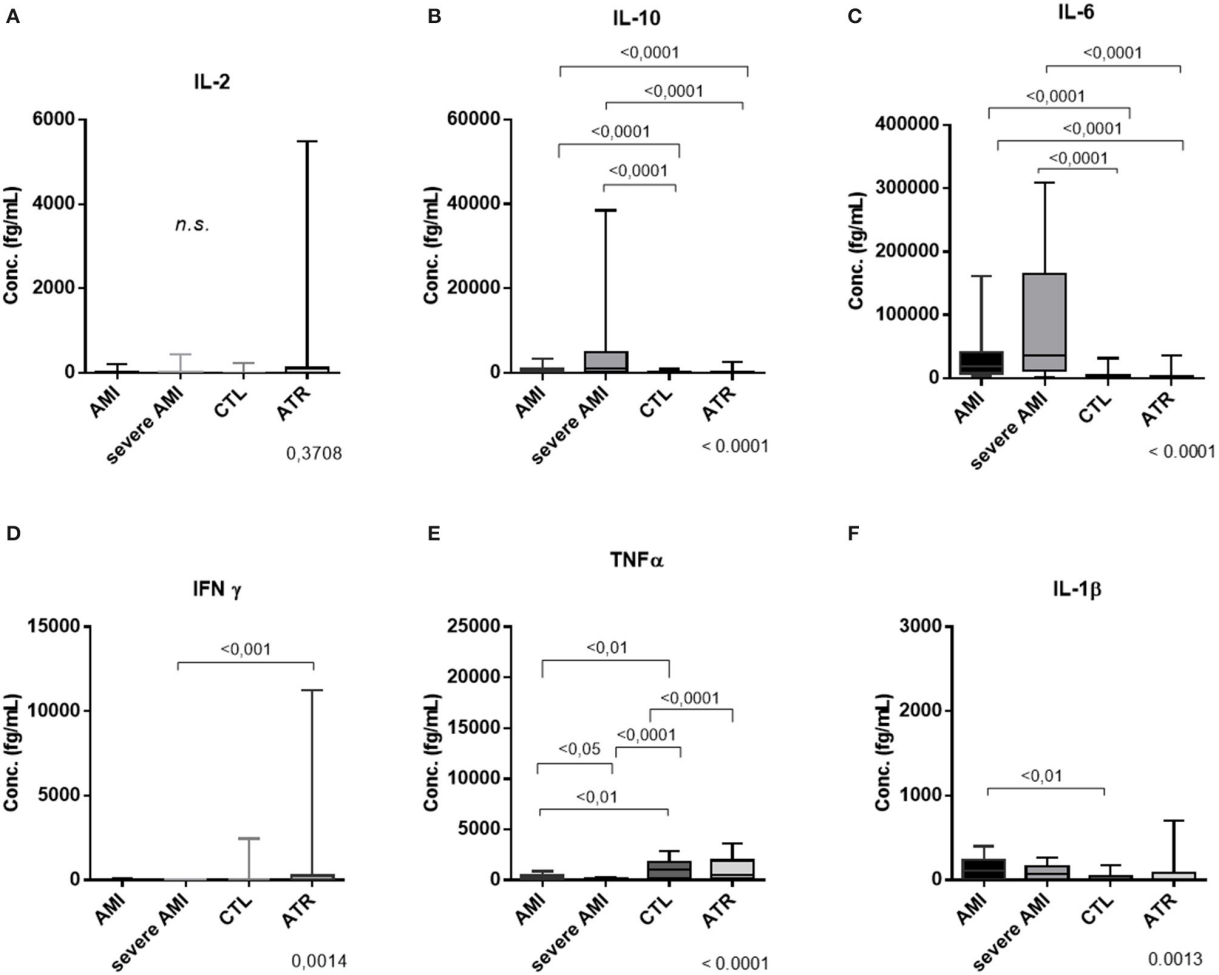
Concentrations of cytokines (fg/mL) in the sera of patients in the different study groups—analysis performed by flow cytometry (CBA). The progression of atherosclerotic disease and the severity of the infarction promote an inflammatory response exacerbated mainly by the IL-6 and IL1B pathways, but the compensation mechanism by IL-10 is not sufficient to contain the inflammation. **(A)** IL-2, **(B)** IL-10, **(C)** IL-6, **(D)** IFN-γ, **(E)** TNFα, **(F)** IL-1β. (AMI *n* = 40, severe AMI *n* = 34, CTL *n* = 34, ATR *n* = 40) (data are expressed as median and interquartile range 25–75%). Comparison by Kruskal-Wallis with Dunn applied as a post-test.

The cytokine panel showed no difference in IL-2 (Figure 7A) among the groups (Kruskal-Wallis, *p* = 0.3708) but greater amounts (median and interquartile ranges 25–75%) of IL-10 in the severe AMI [1,515 (530.9–7,497)] and AMI [385.6 (249.2–913.4)] groups than in the CTL [96.45 (43.79–212.5)] and ATR [95.25 (60.13–158.7)] groups (Kruskal-Wallis, *p* < 0.0001; Figure 7B).

IL-6 exhibited a similar profile, with higher amounts in the AMI [18,632 (7,610–41,591)] and severe AMI [1,15,455 (16,298–1,97,428)] groups than in the CTL [1,349 (787–3,025)] and ATR [2,304 (926.4–4,074)] groups (Kruskal-Wallis, *p* < 0.0001; Figure 7C). The IFγ level of the CTL group [263.6 (0.0–11,645)] was increased compared to that of the severe AMI group [0.0 (0.0–0.0)] (Kruskal-Wallis, *p* < 0.001; Figure 7D). TNFα was more abundant in the CTL [519 (154.5–1,947)] and ATR [1,044 (207.2–1,826)] groups than in the AMI [204.7 (39.95–468.4)] and severe AMI [60.12 (13.91–227.9)] groups (Kruskal-Wallis, *p* < 0.0001; Figure 7E). In addition, the AMI group also exhibited a greater amount of TNFα than the severe AMI group. The IL-1β level was higher in the AMI group [109.7 (0.0–240.5)] than in the ATR group [0.0 (0.0–45.89)] (Kruskal-Wallis, *p* = 0.0013; Figure 7F).

## Discussion

An atherosclerotic plaque may contain proinflammatory cytokines, reactive oxygen species, high levels of oxidized low-density lipoproteins (oxLDL), cholesterol crystals and a variety of damage-associated molecular patterns (8) due to damage to proteins, lipids and DNA, affecting cellular metabolic activity (9). This microenvironment seems to be the main critical predictor for the risk of plaque rupture and thrombosis (10, 11, 39) and infection might participate (40). Different microbiomes may explain a chronic inflammatory state of atherosclerosis, with a more pathogenic microbiome related to more inflammation and greater disease severity (41). Among the pathogens identified in human ruptured thrombosed atherosclerotic plaques, our group described large amounts of *Chlamydophila pneumoniae, Mycoplasma pneumoniae* (42) and other bacteria, where LPS and heat shock proteins stimulate the production of proinflammatory mediators (43). Subsequently, clinical studies confirmed the importance of these bacteria, showing increased levels of anti-*C. pneumoniae* and anti-*M. pneumoniae* antibodies after AMI (44, 45). Our group also observed the presence of EVs coated by a double membrane with electron-lucent content, presenting archaeal DNA, as part of the microbiome in vulnerable plaques and associated with myxomatous degeneration (12).

As already mentioned, archaea, despite being often considered non-pathogenic, are resistant to antibiotic therapy, and induce inflammation via activation of CD8+ T lymphocytes. In addition to promoting metalloproteinase synthesis and oxidation due to its electronegative membrane (15). Our results using morpho-molecular techniques detected increased numbers of large MVs (0.8–1.34 nm) with highly negative surface charge that were positive for archaeal DNA, *Mycoplasma pneumoniae* antigens and MMP9 in the sera of severe myocardial infarction patients, strongly favoring our hypothesis that pathogenic archaea may play a role in worst outcomes of atherosclerosis.

A notable feature of archeal membranes is the ability of the S-layer proteins that make up the membrane to group into a two-dimensional crystalline protein, forming pores that vary from 2 to 8 nm in diameter and can occupy up to 70% of the cellular envelope, becoming permeable depending on the environment in which it is inserted (46). This finding reinforces our hypothesis that the large MVs positive for MMP9 and *M. pneumoniae* antigen identified by Flow Cytometry are Archaea, since they are morphologically similar to a double membrane electron lucent microvesicles and we identified it through hybridization of its DNA.

Archaea and bacteria also release microvesicles (47). EVs are usually considered protective, promoting the removal of harmful cellular compounds and leading to cell survival, but have been described as deleterious, favoring the proliferation of cancer cells, delivering pathogens to uninfected cells and contributing to the development of heart failure (26, 48). Studying Chagas disease, we observed that heart failure in chagasic patients was directly associated with an increased quantity of EVs containing DNA and archaeal collagenase in serum, strong evidence that collagenase-producing archaea play a role in the development of worse clinical outcomes in cardiovascular disease (14).

Due to the low mutation rates of the 16S rRNA region (component of the small 30S subunit of prokaryotic ribosomes) they have been widely used for bacterial identification. However, established primers that specifically amplify the 16S ribosomal gene region of Archaea, a primitive microorganism, are scarce compared to the variety of primers that target bacterial sequences (49). In recent years with the sequencing of the metagenome, the number of phyla of the Archaea domain has increased to more than 20 (50). Additionally, due to the high conservation and sequence similarity, from 90 to 98%, the result of 16S analysis in closely related strains has been considered imprecise and inconsistent with results obtained by other methods (51). Then, we used a probe designed as specific DNA fragment, and to identify bioproducts such as iMVs containing antigens or coming from *M. pneumoniae*, we use antigen immunolabeling.

Using Nanosight equipment, we found the highest numbers of EVs <100 nm (exosomes) and from 100 to 200 nm in stable atherosclerotic and control healthy groups compared with AMI groups, indicative that these EVs are protective. This finding corroborates our result regarding the labeling of CD63 (exosomes marker) used to prove that our method of isolation of EVs is effective. Since we also observed a similar modulation, with greater expression in the groups with good evolution compared to those with severe infarction.

Exosomes have the function of binding to non-vesicular proteins in each physiological medium, thus decreasing their circulating concentration (52, 53). In this study, we also detected a modulation in quantification by Electron Microscopy similar to that observed by Nanosight, giving reliability to this semi-quantitative analysis. It was also possible to observe a greater number of exosomes by this technique microscopy that contained *M. pneumoniae* and MMP9 antigens (using immunoelectron microscopy) in the healthy and stable atherosclerotic group, suggesting that these exosomes can both trap infectious antigens and degrade active MMP9, protecting against the development of rupture of the plaque.

We also believe that the symbiosis between *M. pneumoniae* and archaea in vulnerable plaques leads to the release of iMVs smaller than 200 nm in the peripheral blood, which may play a role in the reduction of protective EVs, explaining zymography data of increased enzyme activity of MMP9 in the sera of infarcted patients, especially those with greater disease severity, compared with healthy and stable atherosclerotic individuals. According to our results, the increase in MMP9 is directly associated with plaque instability, greater infarction, impaired left ventricular function, and fatal clinical evolution in acute myocardial infarction with ST elevation (54–56). A recently published study reported that hypertensive patients who evolved with AMI had erythrocyte deformity with decreased red cell zeta potential and presented lipid peroxidation compared to hypertensive patients who did not have infarcts (57). Additionally, there are descriptions that certain species of gram-negative bacteria in periodontitis patients activate platelets contributing to atherothrombosis (58) and that infection promotes increases in proinflammatory and procoagulant microvesicles derived from the activated host cell membrane (59). There is also evidence that endothelial MVs contain MMP2 and MMP9, in both active and proenzyme forms, and therefore are able to promote matrix degradation. Furthermore, the endogenous inhibitors of MMPs, TIMP-1, and TIMP-2, are present in the same MVs and can thus exert a regulatory effect on the activity of the protease (60).

Regarding inflammation, our present work shows that the typically inflammatory cytokines IL1β, IL-10, and IL-6 are present in higher levels in the sera of infarcted patients, especially in severe cases, compared to healthy and stable atherosclerotic individuals, in accordance with already published studies that suggest that the increases in these cytokines play critical roles in the activation of post-infarction inflammation, promoting matrix degradation, delayed healing of the infarcted area, and cardiomyocyte apoptosis, thus mediating post-AMI dysfunction (61–63). In addition, there is evidence that the simultaneous elevation of IL-10 and IL-6 distinguishes infarcted patients with worse clinical outcomes and is directly correlated with an increased risk for diastolic dysfunction and death within 6 months after the event (64). Currently, an increasing number of studies suggests that the levels of oxidative stress markers in body fluids correlate with the activity of atherosclerotic disease, reinforcing our findings (65, 66).

Interestingly, our study showed a reduced TNFα serum level in infarcted patients, especially in severe patients, compared to atherosclerotic and healthy individuals. TNFα has an ambiguous function, despite being classically described as a proinflammatory cytokine, as just after the ischemic event, TNF-α is recruited to the injured area, and when combined with its type II receptor, it may form a complex that inhibits the inflammatory reaction and ventricular remodeling after AMI, functioning in a protective manner (67–69).

Conversely, inflammatory cytokines, such as TNF-α or IL-1, stimulate the *in vitro* production of monocytes and endothelial cell MVs, potentiating inflammation and conferring high procoagulant activity (70). Local generation of EVs in the hearts of infarcted rats coordinates cardiac inflammation, increasing the release of inflammatory monocyte chemokines and cytokines and amplifying inflammation through a positive feedback signal (71).

In summary, the release of free radicals by archaea, which are present in greater quantities in vulnerable atherosclerotic lesions, causes a highly harmful effect, inducing exacerbated inflammation, the release of MMP9, plaque rupture, thrombosis and AMI; these archaea are pathogenic, presenting MMPs and releasing nanovesicles that may play a role in the lack of protection of exosomes.

## Conclusion

In human atherosclerosis, a microbiome with pathogenic archaea is associated with increased free radical formation, MMP9 release, and increased plaque vulnerability, leading to acute myocardial infarction with a worse prognosis. Co-infection with *M. pneumoniae* leads to the suppression of the immune system and a decrease in protective exosomes. Thus, the present pioneering work demonstrates that the morphomolecular characterization and quantification of EVs and iEVs in serum (archaeal DNA, MMP9+, negative membrane surface charge) constitute a promising way to develop a new biomarker of clinical outcome in CAD. This is an initial study for which the results need to be confirmed with greater patient numbers.

## Data Availability Statement

The original contributions generated for the study are included in the article/Supplementary Material, further inquiries can be directed to the corresponding author/s.

## Ethics Statement

The studies involving human participants were reviewed and approved by Ethics Committee for Research Project Analysis (CAPPesq) of Hospital das Clínicas, Faculty of Medicine, University of São Paulo (HCFMUSP), under protocol numbers 029/04 and SDC 2356/03/150 and also had the approval by CaPPesq from the University Hospital of the University of São Paulo (protocol: 866/08F and SDC: 2356/03/150). The patients/participants provided their written informed consent to participate in this study.

## Author's Note

This manuscript was based on the doctoral thesis of CM.

## Author Contributions

CM, JR, PL, AS, LO, RI, JK, JP, MR, and MH conceived, designed, and performed the experiments. All co-authors participated in the formulation of the manuscript or critically reviewed contributing to its intellectual content, approved the version to be published and agreed to be responsible for all aspects of the work, ensuring that issues related to the accuracy or integrity of any part of the work were properly investigated and resolved.

## Conflict of Interest

The authors declare that the research was conducted in the absence of any commercial or financial relationships that could be construed as a potential conflict of interest.

## Publisher's Note

All claims expressed in this article are solely those of the authors and do not necessarily represent those of their affiliated organizations, or those of the publisher, the editors and the reviewers. Any product that may be evaluated in this article, or claim that may be made by its manufacturer, is not guaranteed or endorsed by the publisher.
