# Supplementary material for: Morphomolecular Characterization of Serum Nanovesicles From Microbiomes Differentiates Stable and Infarcted Atherosclerotic Patients
Source: Front Cardiovasc Med. 2021 Aug 5;8:694851. doi: 10.3389/fcvm.2021.694851 (PMC8375156; doi:10.3389/fcvm.2021.694851)
Supplement: Supplementary file 1 [file Table_1.DOCX]

Supplementary Material

# Supplementary Table

**Supplementary Material – S1**

**Baseline characteristics of the 168 patients studied in the different groups**

| **Patients Characteristics** | **AMI** | **Severe AMI** | **CTL** | **ATR** |
| --- | --- | --- | --- | --- |
|  | **n=40** | **n=34** | **n=34** | **n=40** |
| Age (years) *mean ± standard deviation* | 55.7 ±11.23 | 68.50 ±15.46 | 51.50 ±11.77 | 70.23 ±12.96 |
| Male gender  *(n), %* | (30) 75 | (17) 50 | (23) 67.64 | (29) 72.5 |
| Smokers  *(n), %* | (18) 45 | (13) 38.23 | (3) 8.82 | (17) 42.5 |
| Diabetics  *(n), %* | (18) 45 | (12) 35.29 | N/A | (27) 67.5 |
| Hypercholesterolemics *(n), %* | (29) 72.5 | (20) 58.82 | N/A | (38) 95 |
| Hypertriglyceridemics *(n), %* | (15) 37.5 | (16) 47.05 | N/A | (28) 70 |
| Sedentary  *(n), %* | (13) 32.5 | (26) 76.47 | (7) 20.58 | (15) 37.5 |
| Hypertensive  *(n), %* | (29) 72.5 | (30) 88.23 | N/A | (36) 90 |

| BMI>30  *(n), %* | (14) 35 | (13) 38.23 | (13) 38.23 | (15) 37.5 |
| --- | --- | --- | --- | --- |
| Death  *(n), %* | N/A | (16) 47.05 | N/A | N/A |

**Table S1.** Results are given as mean (SD) or number (%) of subjects. All values of the characteristics, except age are expressed in number of patients *(n)* and percentage. N/A: not applicable
